# Influenza D Virus in Cattle, France, 2011–2014

**DOI:** 10.3201/eid2102.141449

**Published:** 2015-02

**Authors:** Mariette F. Ducatez, Claire Pelletier, Gilles Meyer

**Affiliations:** French National Institute for Agronomy Research (INRA) and Ecole Nationale Vétérinaire (INP-ENVT), Toulouse, France (M.F. Ducatez, G. Meyer);; Laboratoire Départemental d'Analyses (LDA71), Département de Saône-et-Loire, Mâcon, France (C. Pelletier)

**Keywords:** influenza D virus, bovine, France, cattle, viruses

## Abstract

A new influenza virus, genus D, isolated in US pigs and cattle, has also been circulating in cattle in France. It was first identified there in 2011, and an increase was detected in 2014. The virus genome in France is 94%–99% identical to its US counterpart, which suggests intercontinental spillover.

Recent studies in the United States have identified a new genus within the family *Orthomyxoviridae*, tentatively named *Influenzavirus D* (*1*). The new pathogen, C/swine/Oklahoma/1334/2011 (C/OK), was first identified in pigs with influenza-like illness and was only moderately related to previously characterized influenza C viruses (≈50% overall homology between C/OK virus sequence and its closest related sequences). Like human influenza C virus, C/OK harbored 7 genomic segments, whereas influenza A and B viruses have 8. C/OK virus genome was more distant from influenza C virus genomes than influenza A genomes are from influenza B virus genomes. In hemagglutination-inhibition assays, cross-reactivity between antibodies against C/OK virus and human influenza C virus was lacking, which again suggests a new genus in the family *Orthomyxoviridae* (*2*). C/OK-like viruses were also isolated from cattle in the United States in 2013 (*1*) and China in 2014 (*2*).

C/OK was shown to replicate in ferrets, the animal model of choice for studying influenza virus in humans, suggesting that humans could be infected with C/OK-like viruses. Thus, the host range and geographic distribution of C/OK-like viruses (influenza D virus) needs to be investigated. Because cattle have been suggested as the reservoir for this novel influenza virus (*1*), we screened bovine samples in France for influenza D virus and characterized the virus from positive specimens.

## The Study

Bovine lung fragments, deep nasal swab specimens, and trans-tracheal aspiration liquids were submitted to the Laboratoire Départemental d’Analyses de Saône-et-Loire (Mâcon, France) and tested for classical respiratory pathogens. The Unité Mixte de Recherche, Interactions Hôtes—Agents Pathogènes 1225 (Toulouse, France), received and tested 134 samples by using real-time reverse transcription PCR for influenza D virus as previously described (*3*). We tested 25 archived samples per year for 2010–2013 and 34 samples collected during January–March 2014. Six (4.5%) were positive for influenza D virus: 1 each in 2011 and 2012 and 4 in 2014; cycle threshold (C_t_) values ranged from 15 to 35 (Table 1). Co-infections were detected with *Pasteurella multocida*, *Mannheimia haemolytica*, *Histophilus somni*, bovine respiratory syncytial virus, and/or bovine herpesvirus 1 in 4 of the influenza D–positive specimens. Two samples (nos. 5831 and 5920, collected in 2014) were negative for all tested respiratory pathogens, despite reports of clinical signs in the animals (Table 1).

**Table 1:** Cattle samples positive for influenza D virus genome by real-time reverse transcription PCR, France*

Sample ID	Collection date	Sample type	Age	Antimicrobial treatment	Vaccination†	Duration of respiratory signs	FluD (C_T_)	BRSV	BPIV-3	*Pasteurella multocida*	*Mannheimia haemolytica*	*Mycoplasma bovis*	*Histophilus somni*	BCoV	BoHV-1
5731	2014 Jan 25	Lung	Adult	Yes	NC	NC	P (35)	N	N	WP	WP	N	P	NT	P
5831	2014 Feb 5	Lung	12 mo	Yes	Yes	3–4 wk	P (31)	N	N	N	N	N	N	NT	NT
5920	2014 Feb 18	Lung	1 mo	Yes	Yes	3 d	P (27)	N	N	N	N	N	N	N	NT
6106	2014 Mar 14	Lung	1 mo	No	Yes	1 d	P (35)	N	N	WP	N	N	WP	N	NT
2364	2011 Mar 30	Lung	NC	NC	NC	NC	P (35)	NT	NT	P	N	N	N	NT	NT
2986	2012 Jan 17	Nasal swab	NC	NC	NC	NC	P (15)	P	N	WP	WP	N	N	NT	NT

*C_t_, cycle threshold; BCoV, bovine coronavirus; BoHV-1, bovine herpesvirus I; BPIV3, bovine parainfluenzavirus 3; BRSV, bovine respiratory syncytial virus; FluD, influenza D virus; ID, identification; N, negative; NC, not communicated; NT, not tested; P: positive (C_t_<35), WP: weakly positive (35<C_t_<40). †Against BRSV and BPIV-3.

The specimen with the lowest C_t_ value, D/bovine/France/2986/2012 (C_t_ 15) was selected for further molecular characterization, and its full genome was amplified by PCR (primers in Table 2) and sequenced on a 3130XL Applied Biosystems capillary sequencer (Applied Biosystems, Foster City, CA, USA). The sequences were submitted to EMBL (LN559120–LN559126).

**Table 2:** Primer sets used for PCR amplification and sequencing of the full genome of D/bovine/France/2986/2012

Gene	Forward primer, 5′ → 3′	Reverse primer, 5′ → 3′
Hemagglutinin-esterase	FluD_HE-1F: AGCATAAGCAGGAGATTTTCAAAG	FluD_HE-745R: GCACTACATGCTTGTTGC
FluD_HE-667F: GTTTGTGGGACTGAGCAATC	FluD_HE-1350R: CCCTGCTTGCGGTATTATC
FluD_HE-1267F: CCCAAGTATGGCAGATG	fluD_HE-2042R: GCAAGGAGATTTTTTCTAAGATT
Matrix	FluD-MP-8F: GCAGAGGATATTTTTGACGC	FluD-MP-670R: CCCATATGCTATTCTTGCCAG
	FluD-MP-602F: AAAAAAGAGGCCCAGGCAC	FluD-MP-1212R: GCAAGAGGATTTTTTCGCG
Nucleoprotein	FluD-NP-1F: GGCATAAGCAGGAGATTATTAAGC	FluD-NP-949R: TAAAGGCTCTTACTCCAGAATA
	FluD-NP-849F: GCCTTGGTCAATGTGGCTG	FluD-NP-1717R: GGGGACTGCAACAGAACCA
Nonstructural protein	FluD-NS-8F: GCAGGGGTGTACAATTTCAAT	FluD-NS-804R: TCGAAACTGACTTGATTTCATCC
Polymerase 3	FluD-P3–1F: GGCATAAGCAGGAGATTTA	FluD-P3–759R: TTTTCTTCTAGATGTTCCAGTTTGA
	FluD-P3–677F: AAAAGAAATCAGGCTGAATGC	FluD-P3–1467R: CCAAACAAACAGTCAGTTGA
	FluD-P3–1394F: CCCGGAAAGGTCAAGATAG	FluD-P3–2184R: GGAGATTTTTAACATTACAAGGCC
Polymerase basic 1	FluD-PB1–1F: GGCATAAGCAGAGGATTTTAT	FluD-PB1–736R: TTTTCCTCTTTCTCCGTC
	FluD-PB1–631F: AAAAATGAAGTCTCCAACATTG	C/OK-Rev (*2*)
	C/OK-Fwd (*2*)	FluD-PB1–2317R: GATTTTTCTGTTATTAAACAACGC
Polymerase basic 2	FluD-PB2–1F: GGCATAAGCAGAGGATGTC	FluD-PB2–882R: CCCTTATCTTCTCTGCTGG
	FluD-PB2–796F: AAAAGAAGAGAGATGTTAGAGC	FluD-PB2–1776R: TTTTACCCATTATCAAAGCAGG
	FluD-PB2–1724F: GAAAGAATAAACACTGATGATG	FluD-PB2–2353R: GAGGATTTTTTCAATGTGCTTC

The 7 gene segments of D/bovine/France/2986/2012 clearly clustered with US influenza D strains from pigs and cattle (C/OK, C/bovine/Minnesota/628/2013, C/bovine/Minnesota/729/2013, and C/bovine/Oklahoma/660/2013) (Figure), which suggests a common origin of these new influenza viruses. We found no evidence of reassortment between influenza C and D (C/OK-like) viruses. In addition, the splicing pattern of the matrix gene segment and the reduced 5-N-acetyl binding pocket in the hemagglutinin-esterase (HE) protein of D/bovine/France/2986/2012 was similar to that of C/OK and different from that of human influenza C virus, confirming the similarity of D/bovine/France/2956/2012 and the newly described swine and bovine US influenza D virus strains. The estimated ranges of evolutionary distances (in number of substitutions per site using the maximum composite likelihood model) between D/bovine/France/2986/2012 and the 4 US influenza D viruses ranged from 0.8 to 5.7% and were as follows: 1.9%–2.1%, 0.8%–0.9%, 2.1%–2.5%, 2.3%–2.7%, 1.8%–3.8%, 3.6%–4.2%, and 5.1%–5.7% for polymerase basic (PB) 2, PB1, polymerase 3/polymerase acidic, nucleoprotein, matrix, nonstructural protein, and HE gene segments, respectively.

**Figure:**
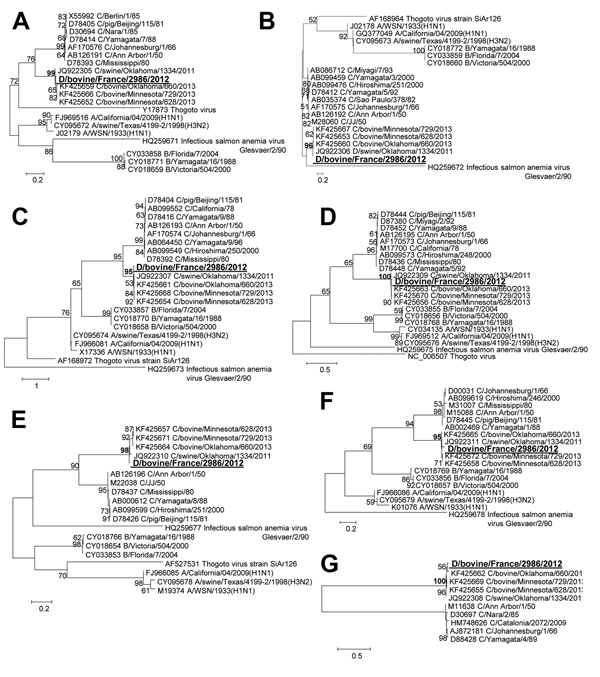
Phylogenetic trees of the 7 gene segments of D/bovine/France/2986/2012 influenza virus at the nucleotide level. A) PB2. B) PB1. C) P3/PA. D) Nucleoprotein E) P42/Matrix. F) Nonstructural protein. G) Hemagglutinin-esterase. Maximum-likelihood analysis with 500 bootstrap replicates (bootstrap values >75 are indicated on the tree nodes). The gene sequences of D/bovine/France/2986/2012 (in large bold underlined font) were compared with representatives of all the *Orthomyxoviridae* genera: all the viral strains used in (*1*). P, polymerase, nucleoprotein, PB, polymerase basic  Scale bars indicate nucleotide substitutions per site.

We also identified unique features in D/bovine/France/2956/2012 genome. Forty unique amino acid substitutions were identified throughout the genome, but the limited available data on influenza D genomes make a functional interpretation of the substitutions difficult to determine. In addition, although the HE proteins of human influenza C and C/OK viruses contain 7 and 6 potential glycosylation sites, respectively, D/bovine/France/2986/2012 has just 5: at positions 28, 54, 146, 346, and 613 (ATG numbering), identical to 5 of the 6 identified for C/OK virus. For influenza A viruses, modifications of N-linked glycosylation sites in the globular head of the hemagglutinin protein have been linked to changes in virulence, antigenicity, receptor-binding preference, fusion activity, and immune evasion (*4*). For example, an increase in glycosylation site numbers has been associated with early stages of influenza A(H1N1) virus evolution, and changes in the positional conversion of the glycosylation sites have been associated with later evolutionary stages of the virus (*5*). The missing potential glycosylation site in D/bovine/France/2986/2012 is located at position 513, probably likely in the F3 = HE2 fusion domain of the protein (*6*) and not in the globular head of the protein. Thus speculating on the putative time course of virus emergence between C/OK-like and D/bovine/France/2986/2012-like strains is difficult. Further studies are needed to understand the phenotype(s) associated with aa substitutions in influenza D viruses.

## Conclusions

Webster et al. suggested the existence of a common ancestor for influenza A, B, and C viruses and a more recent common ancestor to influenza A and B viruses only considering the different genome organizations between influenza A/B and influenza C viruses (*6*). Sheng et al. recently estimated the time of divergence between influenza C and D virus gene segments at 334 years for PB1 to 1,299 years for HE (*7*). The time of emergence and evolutionary rate of influenza D viruses need to be examined as more data become available. A puzzling question raised by our current study is the geographic origin of influenza D strains: were cattle in France contaminated by their North American counterparts or vice versa? Did co-evolution occur? Did the pathogen originate from a distinct location or from a distinct host?

Retrospective studies with archived samples would help date the emergence of influenza D viruses and enable an understanding of their evolution. In addition, the geographic prevalence still needs to be investigated.

The pathogen may have spread to swine and cattle in recent years only; efforts should be made to find the virus host range and its reservoir species and to evaluate the public health relevance of this new pathogen. Finally, surveillance projects with larger cohorts, as well as experimental infections, need to be conducted before 1) the causality between respiratory symptoms and influenza D virus infection in cattle can be established, 2) recommendations on samples to collect can be given, and 3) prevalence can be compared in different geographic areas. Although the causative agent(s) of some respiratory infections in the field remain(s) unknown (G. Meyer, pers. comm.) and although 2 of the positive specimens in our study originated from young cattle with respiratory symptoms but no identified respiratory pathogen, further studies are also warranted to provide an understanding of the pathobiology of influenza D virus in cattle and its putative role in complex bovine respiratory disease.
